# The Cincinnati College of Medicine and Surgery

**Published:** 1870-07

**Authors:** 


### Editorial.

### THE CINCINNATI COLLEGE OF MEDICINE AND SURGERY.

The announcement of this college is on our table. It is a pretty thing. The college has a good Board of Trustees, and an able Faculty. We have the pleasure of a personal acquaintance with some of the professors; and they can tell all they *profess* to; and the others are probably brighter even than they; for it is not our usual luck to be acquainted with the smartest men. The college has ten professors, a lecturer, and a demonstrator. The session lasts between three and four months; and the instruction is not only thorough, but dog-cheap; that is, the tickets for the entire course cost exactly as much as a moderately well bred black-and-tan pup. Now, if you can't afford two invaluable luxuries, try to get along without the dog, and take the tickets. We would, if we had time. Think of one of those learned men talking to you three or four months for $2.50! You'll pay Wendell Phillips $1.00 for saying his piece about "lost arts," when there are no lost arts; but here you will be told about *found* arts (and sciences, too) at the rate of less than ten cents a say.

Men who have no thought of practicing either medicine or surgery, ought to attend a course, at least. Anatomy, physiology, chemistry, toxicology, jurisprudence, and psychology are profitable for anybody; and they can all be learned here for the price of an average pup. Medical graduates can post up here with more ease and less expense than by any other plan; and that is the reason our classmate, Doctor Gerwe, is found in the list of students for 1869–70. Though he graduated in 1849 he is a *student* yet; and it is handy to have such a man in a class. If the *teacher* is at a loss, he can ask the student. We hope to see scores of practitioners following his example.

This college has a lecturer on "Dental Medicine and Surgery." The announcement says: "The Faculty, feeling the importance of some knowledge of dental medicine and surgery to the country physician, have associated with them Dr. J. G. Willis, a practical Dentist, as lecturer in that department."

This is not "unmotherly," but as it should be.

